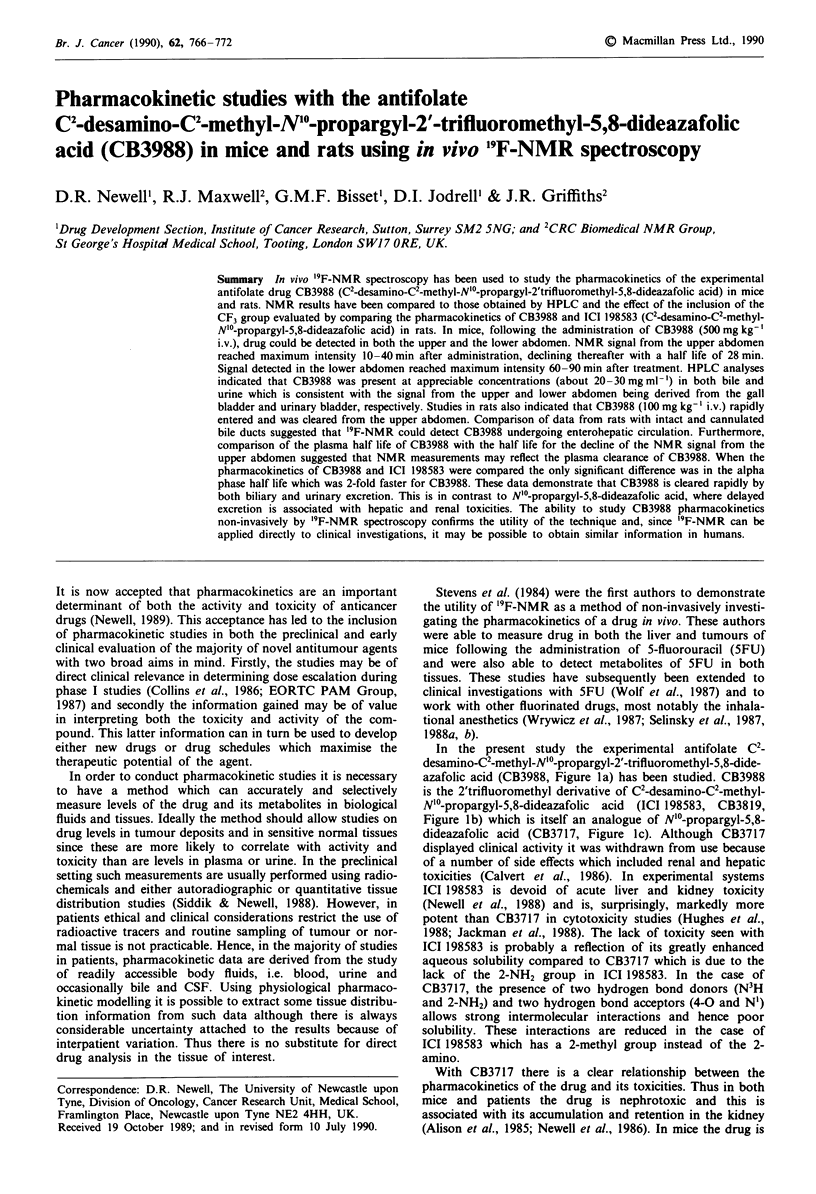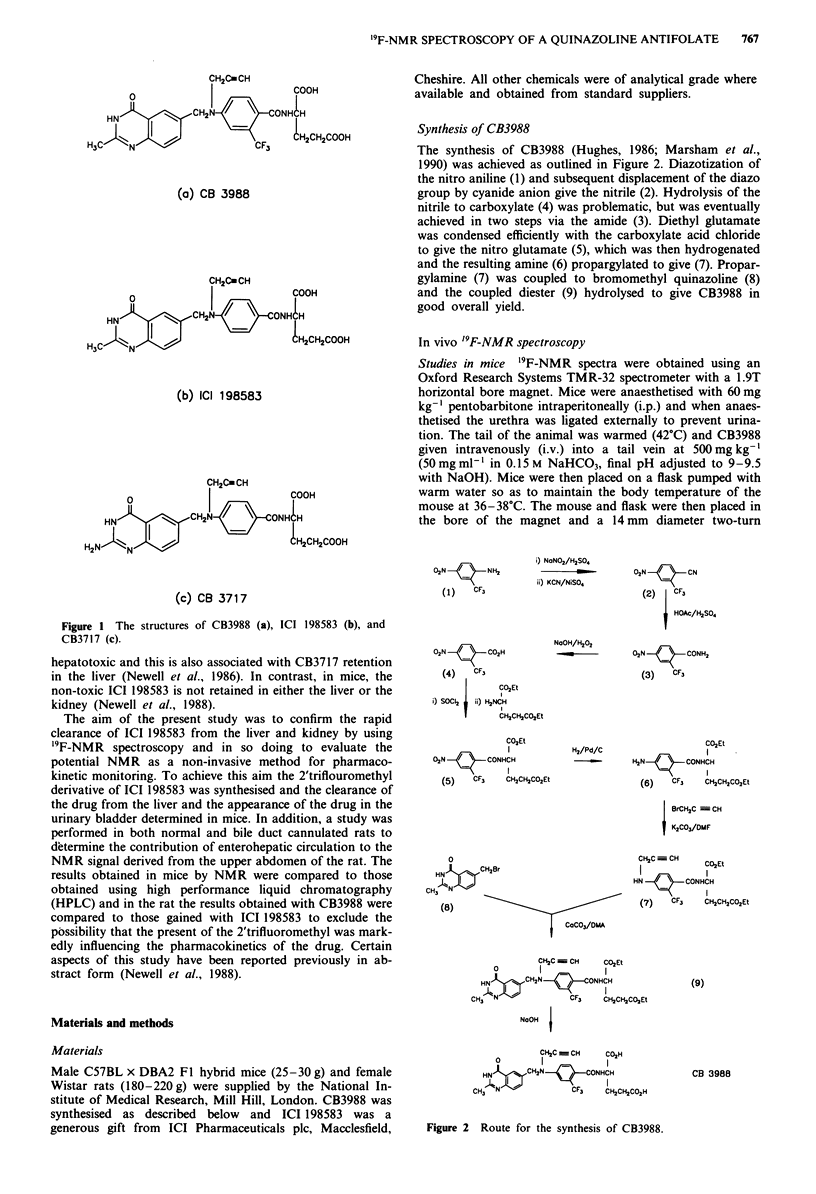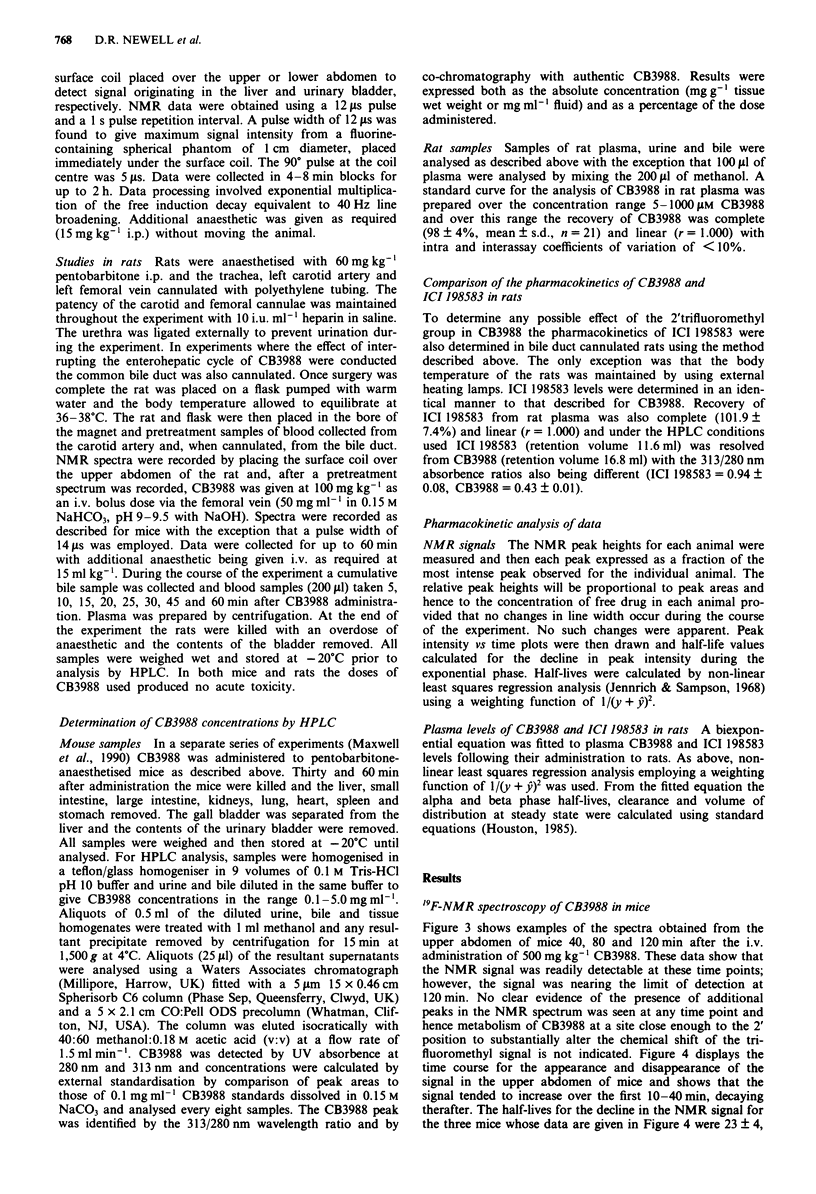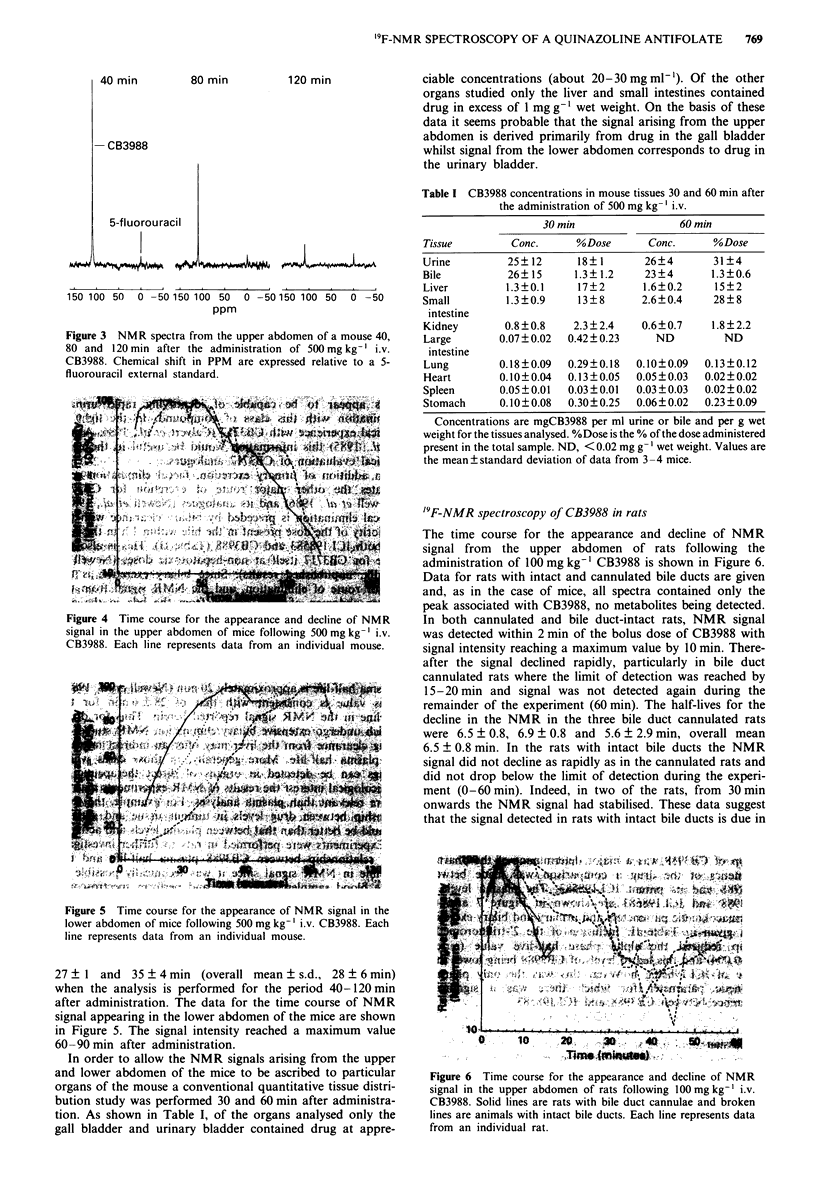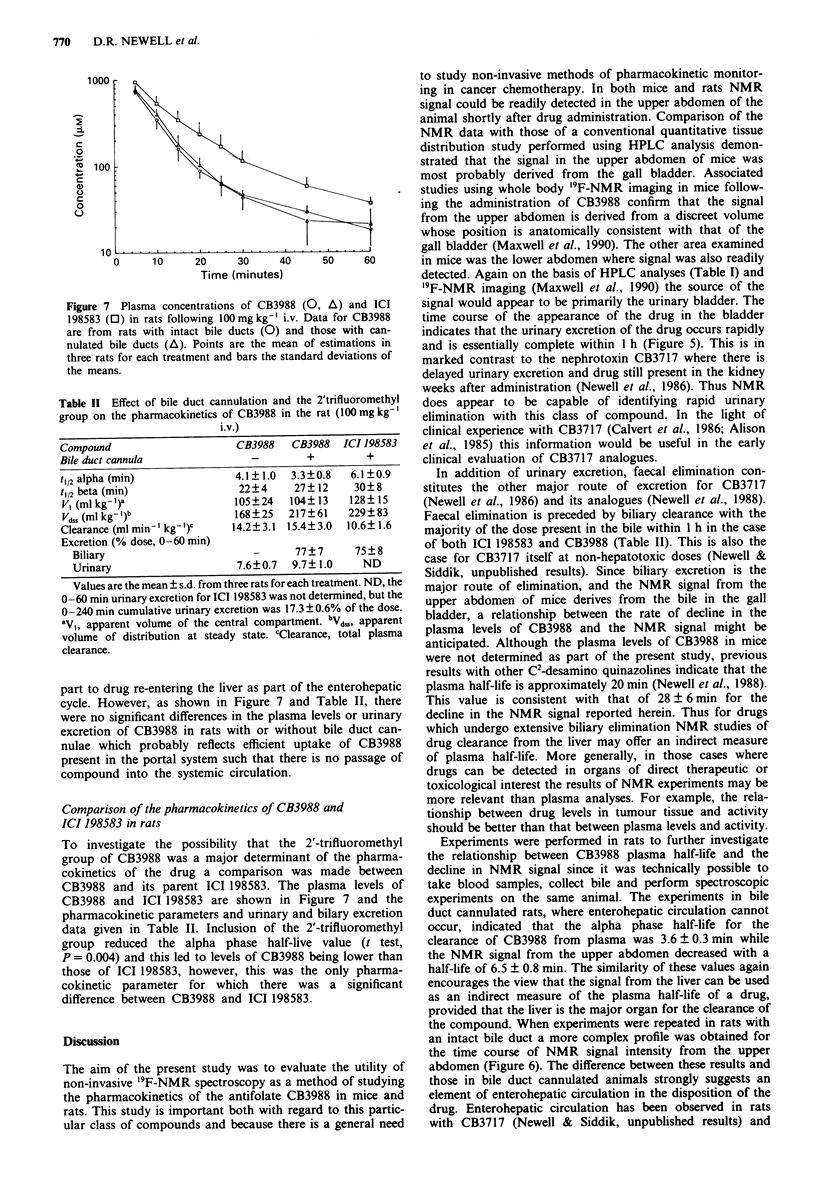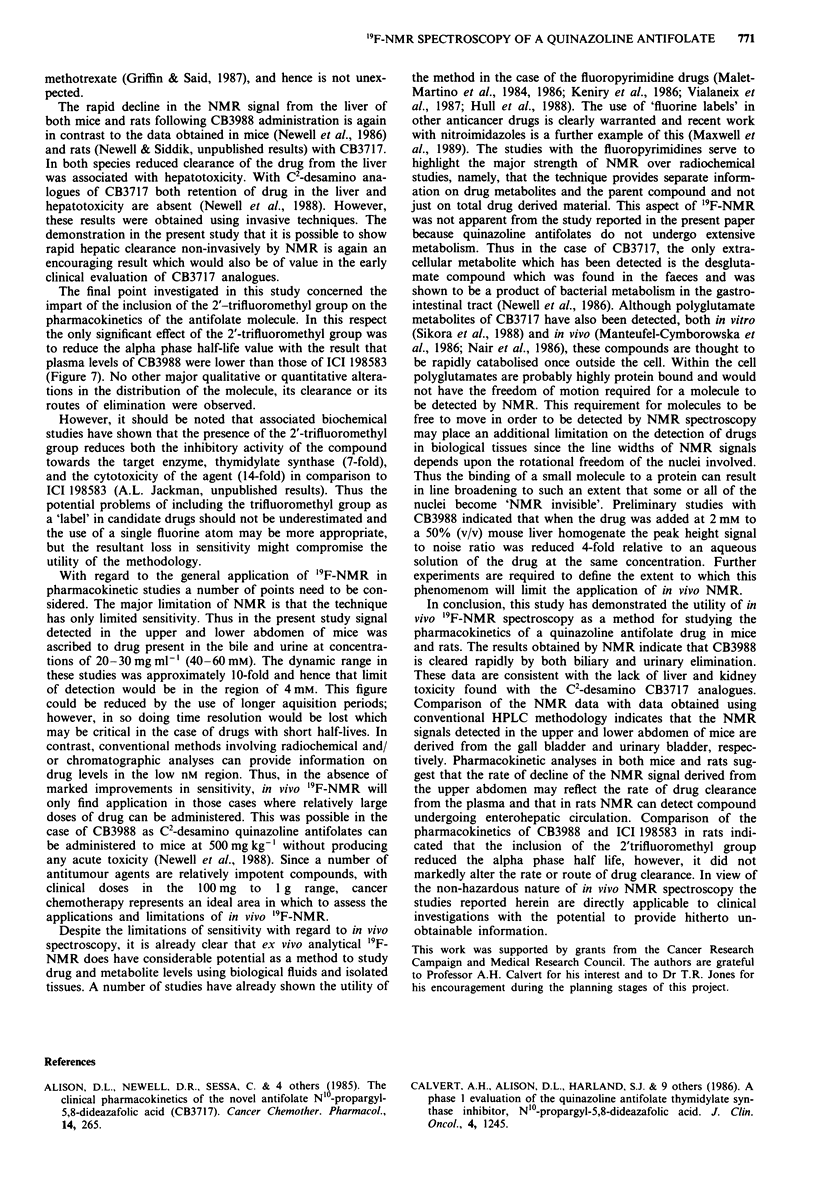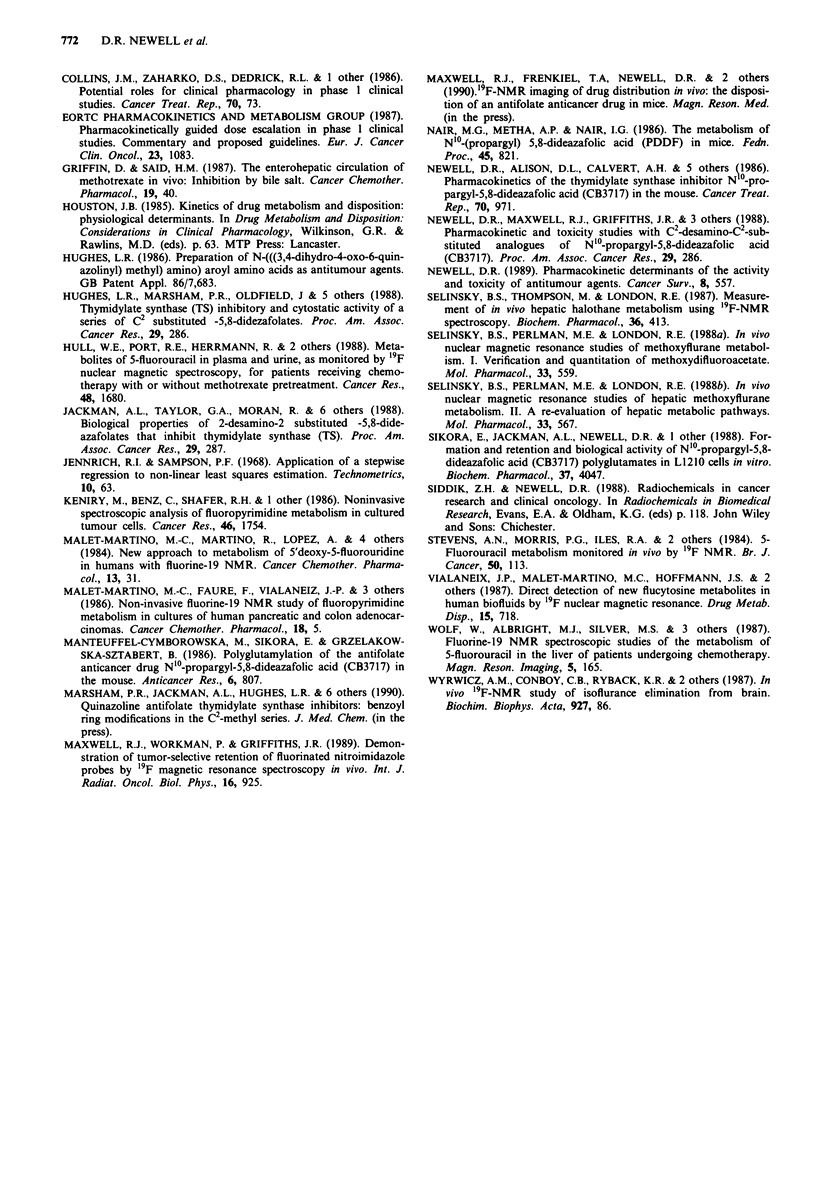# Pharmacokinetic studies with the antifolate C2-desamino-C2-methyl-N10-propargyl-2'-trifluoromethyl-5,8-dideazafolic acid (CB3988) in mice and rats using in vivo 19F-NMR spectroscopy.

**DOI:** 10.1038/bjc.1990.376

**Published:** 1990-11

**Authors:** D. R. Newell, R. J. Maxwell, G. M. Bisset, D. I. Jodrell, J. R. Griffiths

**Affiliations:** Drug Development Section, Institute of Cancer Research, Sutton, Surrey, UK.

## Abstract

In vivo 19F-NMR spectroscopy has been used to study the pharmacokinetics of the experimental antifolate drug CB3988 (C2-desamino-C2-methyl-N10-propargyl-2'trifluoromethyl-5,8-dideazafolic acid) in mice and rats. NMR results have been compared to those obtained by HPLC and the effect of the inclusion of the CF3 group evaluated by comparing the pharmacokinetics of CB3988 and ICI 198583 (C2-desamino-C2-methyl-N10-propargyl-5,8-dideazafolic acid) in rats. In mice, following the administration of CB3988 (500 mg kg-1 i.v.), drug could be detected in both the upper and the lower abdomen. NMR signal from the upper abdomen reached maximum intensity 10-40 min after administration, declining thereafter with a half life of 28 min. Signal detected in the lower abdomen reached maximum intensity 60-90 min after treatment. HPLC analyses indicated that CB3988 was present at appreciable concentrations (about 20-30 mg ml-1) in both bile and urine which is consistent with the signal from the upper and lower abdomen being derived from the gall bladder and urinary bladder, respectively. Studies in rats also indicated that CB3988 (100 mg kg-1 i.v.) rapidly entered and was cleared from the upper abdomen. Comparison of data from rats with intact and cannulated bile ducts suggested that 19F-NMR could detect CB3988 undergoing enterohepatic circulation. Furthermore, comparison of the plasma half life of CB3988 with the half life for the decline of the NMR signal from the upper abdomen suggested that NMR measurements may reflect the plasma clearance of CB3988. When the pharmacokinetics of CB3988 and ICI 198583 were compared the only significant difference was in the alpha phase half life which was 2-fold faster for CB3988. These data demonstrate that CB3988 is cleared rapidly by both biliary and urinary excretion. This is in contrast to N10-propargyl-5,8-dideazafolic acid, where delayed excretion is associated with hepatic and renal toxicities. The ability to study CB3988 pharmacokinetics non-invasively by 19F-NMR spectroscopy confirms the utility of the technique and, since 19F-NMR can be applied directly to clinical investigations, it may be possible to obtain similar information in humans.


*Br. J. Cancer* (1990), **62**, 766–772 © Macmillan Press Ltd., 1990

### Pharmacokinetic studies with the antifolate $C^2$-desamino-$C^2$-methyl-$N^{10}$-propargyl-2′-trifluoromethyl-5,8-dideazafolic acid (CB3988) in mice and rats using *in vivo* $^{19}$F-NMR spectroscopy

D.R. Newell[1], R.J. Maxwell[2], G.M.F. Bisset[1], D.I. Jodrell[1] & J.R. Griffiths[2]

[1]*Drug Development Section, Institute of Cancer Research, Sutton, Surrey SM2 5NG; and* [2]*CRC Biomedical NMR Group, St George's Hospital Medical School, Tooting, London SW17 0RE, UK.*

**Summary** *In vivo* $^{19}$F-NMR spectroscopy has been used to study the pharmacokinetics of the experimental antifolate drug CB3988 ($C^2$-desamino-$C^2$-methyl-$N^{10}$-propargyl-2′trifluoromethyl-5,8-dideazafolic acid) in mice and rats. NMR results have been compared to those obtained by HPLC and the effect of the inclusion of the $CF_3$ group evaluated by comparing the pharmacokinetics of CB3988 and ICI 198583 ($C^2$-desamino-$C^2$-methyl-$N^{10}$-propargyl-5,8-dideazafolic acid) in rats. In mice, following the administration of CB3988 (500 mg kg$^{-1}$ i.v.), drug could be detected in both the upper and the lower abdomen. NMR signal from the upper abdomen reached maximum intensity 10–40 min after administration, declining thereafter with a half life of 28 min. Signal detected in the lower abdomen reached maximum intensity 60–90 min after treatment. HPLC analyses indicated that CB3988 was present at appreciable concentrations (about 20–30 mg ml$^{-1}$) in both bile and urine which is consistent with the signal from the upper and lower abdomen being derived from the gall bladder and urinary bladder, respectively. Studies in rats also indicated that CB3988 (100 mg kg$^{-1}$ i.v.) rapidly entered and was cleared from the upper abdomen. Comparison of data from rats with intact and cannulated bile ducts suggested that $^{19}$F-NMR could detect CB3988 undergoing enterohepatic circulation. Furthermore, comparison of the plasma half life of CB3988 with the half life for the decline of the NMR signal from the upper abdomen suggested that NMR measurements may reflect the plasma clearance of CB3988. When the pharmacokinetics of CB3988 and ICI 198583 were compared the only significant difference was in the alpha phase half life which was 2-fold faster for CB3988. These data demonstrate that CB3988 is cleared rapidly by both biliary and urinary excretion. This is in contrast to $N^{10}$-propargyl-5,8-dideazafolic acid, where delayed excretion is associated with hepatic and renal toxicities. The ability to study CB3988 pharmacokinetics non-invasively by $^{19}$F-NMR spectroscopy confirms the utility of the technique and, since $^{19}$F-NMR can be applied directly to clinical investigations, it may be possible to obtain similar information in humans.

It is now accepted that pharmacokinetics are an important determinant of both the activity and toxicity of anticancer drugs (Newell, 1989). This acceptance has led to the inclusion of pharmacokinetic studies in both the preclinical and early clinical evaluation of the majority of novel antitumour agents with two broad aims in mind. Firstly, the studies may be of direct clinical relevance in determining dose escalation during phase I studies (Collins *et al.*, 1986; EORTC PAM Group, 1987) and secondly the information gained may be of value in interpreting both the toxicity and activity of the compound. This latter information can in turn be used to develop either new drugs or drug schedules which maximise the therapeutic potential of the agent.

In order to conduct pharmacokinetic studies it is necessary to have a method which can accurately and selectively measure levels of the drug and its metabolites in biological fluids and tissues. Ideally the method should allow studies on drug levels in tumour deposits and in sensitive normal tissues since these are more likely to correlate with activity and toxicity than are levels in plasma or urine. In the preclinical setting such measurements are usually performed using radiochemicals and either autoradiographic or quantitative tissue distribution studies (Siddik & Newell, 1988). However, in patients ethical and clinical considerations restrict the use of radioactive tracers and routine sampling of tumour or normal tissue is not practicable. Hence, in the majority of studies in patients, pharmacokinetic data are derived from the study of readily accessible body fluids, i.e. blood, urine and occasionally bile and CSF. Using physiological pharmacokinetic modelling it is possible to extract some tissue distribution information from such data although there is always considerable uncertainty attached to the results because of interpatient variation. Thus there is no substitute for direct drug analysis in the tissue of interest.

Correspondence: D.R. Newell, The University of Newcastle upon Tyne, Division of Oncology, Cancer Research Unit, Medical School, Framlington Place, Newcastle upon Tyne NE2 4HH, UK.
Received 19 October 1989; and in revised form 10 July 1990.

Stevens *et al.* (1984) were the first authors to demonstrate the utility of $^{19}$F-NMR as a method of non-invasively investigating the pharmacokinetics of a drug *in vivo*. These authors were able to measure drug in both the liver and tumours of mice following the administration of 5-fluorouracil (5FU) and were also able to detect metabolites of 5FU in both tissues. These studies have subsequently been extended to clinical investigations with 5FU (Wolf *et al.*, 1987) and to work with other fluorinated drugs, most notably the inhalational anesthetics (Wrywicz *et al.*, 1987; Selinsky *et al.*, 1987, 1988*a*, *b*).

In the present study the experimental antifolate $C^2$-desamino-$C^2$-methyl-$N^{10}$-propargyl-2′-trifluoromethyl-5,8-dideazafolic acid (CB3988, Figure 1a) has been studied. CB3988 is the 2′trifluoromethyl derivative of $C^2$-desamino-$C^2$-methyl-$N^{10}$-propargyl-5,8-dideazafolic acid (ICI 198583, CB3819, Figure 1b) which is itself an analogue of $N^{10}$-propargyl-5,8-dideazafolic acid (CB3717, Figure 1c). Although CB3717 displayed clinical activity it was withdrawn from use because of a number of side effects which included renal and hepatic toxicities (Calvert *et al.*, 1986). In experimental systems ICI 198583 is devoid of acute liver and kidney toxicity (Newell *et al.*, 1988) and is, surprisingly, markedly more potent than CB3717 in cytotoxicity studies (Hughes *et al.*, 1988; Jackman *et al.*, 1988). The lack of toxicity seen with ICI 198583 is probably a reflection of its greatly enhanced aqueous solubility compared to CB3717 which is due to the lack of the 2-$NH_2$ group in ICI 198583. In the case of CB3717, the presence of two hydrogen bond donors ($N^3H$ and 2-$NH_2$) and two hydrogen bond acceptors (4-O and $N^1$) allows strong intermolecular interactions and hence poor solubility. These interactions are reduced in the case of ICI 198583 which has a 2-methyl group instead of the 2-amino.

With CB3717 there is a clear relationship between the pharmacokinetics of the drug and its toxicities. Thus in both mice and patients the drug is nephrotoxic and this is associated with its accumulation and retention in the kidney (Alison *et al.*, 1985; Newell *et al.*, 1986). In mice the drug is

(a) CB 3988

(b) ICI 198583

(c) CB 3717

**Figure 1** The structures of CB3988 (a), ICI 198583 (b), and CB3717 (c).

hepatotoxic and this is also associated with CB3717 retention in the liver (Newell *et al.*, 1986). In contrast, in mice, the non-toxic ICI 198583 is not retained in either the liver or the kidney (Newell *et al.*, 1988).

The aim of the present study was to confirm the rapid clearance of ICI 198583 from the liver and kidney by using $^{19}$F-NMR spectroscopy and in so doing to evaluate the potential NMR as a non-invasive method for pharmacokinetic monitoring. To achieve this aim the 2'triflouromethyl derivative of ICI 198583 was synthesised and the clearance of the drug from the liver and the appearance of the drug in the urinary bladder determined in mice. In addition, a study was performed in both normal and bile duct cannulated rats to determine the contribution of enterohepatic circulation to the NMR signal derived from the upper abdomen of the rat. The results obtained in mice by NMR were compared to those obtained using high performance liquid chromatography (HPLC) and in the rat the results obtained with CB3988 were compared to those gained with ICI 198583 to exclude the possibility that the present of the 2'trifluoromethyl was markedly influencing the pharmacokinetics of the drug. Certain aspects of this study have been reported previously in abstract form (Newell *et al.*, 1988).

### Materials and methods

### *Materials*

Male C57BL x DBA2 F1 hybrid mice (25–30 g) and female Wistar rats (180–220 g) were supplied by the National Institute of Medical Research, Mill Hill, London. CB3988 was synthesised as described below and ICI 198583 was a generous gift from ICI Pharmaceuticals plc, Macclesfield, Cheshire. All other chemicals were of analytical grade where available and obtained from standard suppliers.

### *Synthesis of CB3988*

The synthesis of CB3988 (Hughes, 1986; Marsham *et al.*, 1990) was achieved as outlined in Figure 2. Diazotization of the nitro aniline (1) and subsequent displacement of the diazo group by cyanide anion give the nitrile (2). Hydrolysis of the nitrile to carboxylate (4) was problematic, but was eventually achieved in two steps via the amide (3). Diethyl glutamate was condensed efficiently with the carboxylate acid chloride to give the nitro glutamate (5), which was then hydrogenated and the resulting amine (6) propargylated to give (7). Propargylamine (7) was coupled to bromomethyl quinazoline (8) and the coupled diester (9) hydrolysed to give CB3988 in good overall yield.

### In vivo *$^{19}$F-NMR spectroscopy*

*Studies in mice* $^{19}$F-NMR spectra were obtained using an Oxford Research Systems TMR-32 spectrometer with a 1.9T horizontal bore magnet. Mice were anaesthetised with 60 mg $kg^{-1}$ pentobarbitone intraperitoneally (i.p.) and when anaesthetised the urethra was ligated externally to prevent urination. The tail of the animal was warmed (42°C) and CB3988 given intravenously (i.v.) into a tail vein at 500 mg $kg^{-1}$ (50 mg $ml^{-1}$ in 0.15 M $NaHCO_3$, final pH adjusted to 9–9.5 with NaOH). Mice were then placed on a flask pumped with warm water so as to maintain the body temperature of the mouse at 36–38°C. The mouse and flask were then placed in the bore of the magnet and a 14 mm diameter two-turn

**Figure 2** Route for the synthesis of CB3988.

surface coil placed over the upper or lower abdomen to detect signal originating in the liver and urinary bladder, respectively. NMR data were obtained using a 12 μs pulse and a 1 s pulse repetition interval. A pulse width of 12 μs was found to give maximum signal intensity from a fluorine-containing spherical phantom of 1 cm diameter, placed immediately under the surface coil. The 90° pulse at the coil centre was 5 μs. Data were collected in 4–8 min blocks for up to 2 h. Data processing involved exponential multiplication of the free induction decay equivalent to 40 Hz line broadening. Additional anaesthetic was given as required (15 mg $kg^{-1}$ i.p.) without moving the animal.

*Studies in rats* Rats were anaesthetised with 60 mg $kg^{-1}$ pentobarbitone i.p. and the trachea, left carotid artery and left femoral vein cannulated with polyethylene tubing. The patency of the carotid and femoral cannulae was maintained throughout the experiment with 10 i.u. $ml^{-1}$ heparin in saline. The urethra was ligated externally to prevent urination during the experiment. In experiments where the effect of interrupting the enterohepatic cycle of CB3988 were conducted the common bile duct was also cannulated. Once surgery was complete the rat was placed on a flask pumped with warm water and the body temperature allowed to equilibrate at 36–38°C. The rat and flask were then placed in the bore of the magnet and pretreatment samples of blood collected from the carotid artery and, when cannulated, from the bile duct. NMR spectra were recorded by placing the surface coil over the upper abdomen of the rat and, after a pretreatment spectrum was recorded, CB3988 was given at 100 mg $kg^{-1}$ as an i.v. bolus dose via the femoral vein (50 mg $ml^{-1}$ in 0.15 M $NaHCO_3$, pH 9–9.5 with NaOH). Spectra were recorded as described for mice with the exception that a pulse width of 14 μs was employed. Data were collected for up to 60 min with additional anaesthetic being given i.v. as required at 15 ml $kg^{-1}$. During the course of the experiment a cumulative bile sample was collected and blood samples (200 μl) taken 5, 10, 15, 20, 25, 30, 45 and 60 min after CB3988 administration. Plasma was prepared by centrifugation. At the end of the experiment the rats were killed with an overdose of anaesthetic and the contents of the bladder removed. All samples were weighed wet and stored at −20°C prior to analysis by HPLC. In both mice and rats the doses of CB3988 used produced no acute toxicity.

### *Determination of CB3988 concentrations by HPLC*

*Mouse samples* In a separate series of experiments (Maxwell *et al.*, 1990) CB3988 was administered to pentobarbitone-anaesthetised mice as described above. Thirty and 60 min after administration the mice were killed and the liver, small intestine, large intestine, kidneys, lung, heart, spleen and stomach removed. The gall bladder was separated from the liver and the contents of the urinary bladder were removed. All samples were weighed and then stored at −20°C until analysed. For HPLC analysis, samples were homogenised in a teflon/glass homogeniser in 9 volumes of 0.1 M Tris-HCl pH 10 buffer and urine and bile diluted in the same buffer to give CB3988 concentrations in the range 0.1–5.0 mg $ml^{-1}$. Aliquots of 0.5 ml of the diluted urine, bile and tissue homogenates were treated with 1 ml methanol and any resultant precipitate removed by centrifugation for 15 min at 1,500 *g* at 4°C. Aliquots (25 μl) of the resultant supernatants were analysed using a Waters Associates chromatograph (Millipore, Harrow, UK) fitted with a 5 μm 15 × 0.46 cm Spherisorb C6 column (Phase Sep, Queensferry, Clwyd, UK) and a 5 × 2.1 cm CO:Pell ODS precolumn (Whatman, Clifton, NJ, USA). The column was eluted isocratically with 40:60 methanol:0.18 M acetic acid (v:v) at a flow rate of 1.5 ml $min^{-1}$. CB3988 was detected by UV absorbence at 280 nm and 313 nm and concentrations were calculated by external standardisation by comparison of peak areas to those of 0.1 mg $ml^{-1}$ CB3988 standards dissolved in 0.15 M $NaCO_3$ and analysed every eight samples. The CB3988 peak was identified by the 313/280 nm wavelength ratio and by co-chromatography with authentic CB3988. Results were expressed both as the absolute concentration (mg $g^{-1}$ tissue wet weight or mg $ml^{-1}$ fluid) and as a percentage of the dose administered.

*Rat samples* Samples of rat plasma, urine and bile were analysed as described above with the exception that 100 μl of plasma were analysed by mixing the 200 μl of methanol. A standard curve for the analysis of CB3988 in rat plasma was prepared over the concentration range 5–1000 μM CB3988 and over this range the recovery of CB3988 was complete (98 ± 4%, mean ± s.d., $n = 21$) and linear ($r = 1.000$) with intra and interassay coefficients of variation of < 10%.

### *Comparison of the pharmacokinetics of CB3988 and ICI 198583 in rats*

To determine any possible effect of the 2′trifluoromethyl group in CB3988 the pharmacokinetics of ICI 198583 were also determined in bile duct cannulated rats using the method described above. The only exception was that the body temperature of the rats was maintained by using external heating lamps. ICI 198583 levels were determined in an identical manner to that described for CB3988. Recovery of ICI 198583 from rat plasma was also complete (101.9 ± 7.4%) and linear ($r = 1.000$) and under the HPLC conditions used ICI 198583 (retention volume 11.6 ml) was resolved from CB3988 (retention volume 16.8 ml) with the 313/280 nm absorbence ratios also being different (ICI 198583 = 0.94 ± 0.08, CB3988 = 0.43 ± 0.01).

### *Pharmacokinetic analysis of data*

*NMR signals* The NMR peak heights for each animal were measured and then each peak expressed as a fraction of the most intense peak observed for the individual animal. The relative peak heights will be proportional to peak areas and hence to the concentration of free drug in each animal provided that no changes in line width occur during the course of the experiment. No such changes were apparent. Peak intensity *vs* time plots were then drawn and half-life values calculated for the decline in peak intensity during the exponential phase. Half-lives were calculated by non-linear least squares regression analysis (Jennrich & Sampson, 1968) using a weighting function of $1/(y + \hat{y})^2$.

*Plasma levels of CB3988 and ICI 198583 in rats* A biexponential equation was fitted to plasma CB3988 and ICI 198583 levels following their administration to rats. As above, non-linear least squares regression analysis employing a weighting function of $1/(y + \hat{y})^2$ was used. From the fitted equation the alpha and beta phase half-lives, clearance and volume of distribution at steady state were calculated using standard equations (Houston, 1985).

### Results

### *$^{19}$F-NMR spectroscopy of CB3988 in mice*

Figure 3 shows examples of the spectra obtained from the upper abdomen of mice 40, 80 and 120 min after the i.v. administration of 500 mg $kg^{-1}$ CB3988. These data show that the NMR signal was readily detectable at these time points; however, the signal was nearing the limit of detection at 120 min. No clear evidence of the presence of additional peaks in the NMR spectrum was seen at any time point and hence metabolism of CB3988 at a site close enough to the 2′ position to substantially alter the chemical shift of the trifluoromethyl signal is not indicated. Figure 4 displays the time course for the appearance and disappearance of the signal in the upper abdomen of mice and shows that the signal tended to increase over the first 10–40 min, decaying therafter. The half-lives for the decline in the NMR signal for the three mice whose data are given in Figure 4 were 23 ± 4,

**Figure 3** NMR spectra from the upper abdomen of a mouse 40, 80 and 120 min after the administration of 500 mg kg$^{-1}$ i.v. CB3988. Chemical shift in PPM are expressed relative to a 5-fluorouracil external standard.

**Figure 4** Time course for the appearance and decline of NMR signal in the upper abdomen of mice following 500 mg kg$^{-1}$ i.v. CB3988. Each line represents data from an individual mouse.

**Figure 5** Time course for the appearance of NMR signal in the lower abdomen of mice following 500 mg kg$^{-1}$ i.v. CB3988. Each line represents data from an individual mouse.

27 ± 1 and 35 ± 4 min (overall mean ± s.d., 28 ± 6 min) when the analysis is performed for the period 40–120 min after administration. The data for the time course of NMR signal appearing in the lower abdomen of the mice are shown in Figure 5. The signal intensity reached a maximum value 60–90 min after administration.

In order to allow the NMR signals arising from the upper and lower abdomen of the mice to be ascribed to particular organs of the mouse a conventional quantitative tissue distribution study was performed 30 and 60 min after administration. As shown in Table I, of the organs analysed only the gall bladder and urinary bladder contained drug at appreciable concentrations (about 20–30 mg ml$^{-1}$). Of the other organs studied only the liver and small intestines contained drug in excess of 1 mg g$^{-1}$ wet weight. On the basis of these data it seems probable that the signal arising from the upper abdomen is derived primarily from drug in the gall bladder whilst signal from the lower abdomen corresponds to drug in the urinary bladder.

**Table I** CB3988 concentrations in mouse tissues 30 and 60 min after the administration of 500 mg kg$^{-1}$ i.v.

| | *30 min* | | *60 min* | |
|---|---|---|---|---|
| *Tissue* | *Conc.* | *%Dose* | *Conc.* | *%Dose* |
| Urine | 25±12 | 18±1 | 26±4 | 31±4 |
| Bile | 26±15 | 1.3±1.2 | 23±4 | 1.3±0.6 |
| Liver | 1.3±0.1 | 17±2 | 1.6±0.2 | 15±2 |
| Small intestine | 1.3±0.9 | 13±8 | 2.6±0.4 | 28±8 |
| Kidney | 0.8±0.8 | 2.3±2.4 | 0.6±0.7 | 1.8±2.2 |
| Large intestine | 0.07±0.02 | 0.42±0.23 | ND | ND |
| Lung | 0.18±0.09 | 0.29±0.18 | 0.10±0.09 | 0.13±0.12 |
| Heart | 0.10±0.04 | 0.13±0.05 | 0.05±0.03 | 0.02±0.02 |
| Spleen | 0.05±0.01 | 0.03±0.01 | 0.03±0.03 | 0.02±0.02 |
| Stomach | 0.10±0.08 | 0.30±0.25 | 0.06±0.02 | 0.23±0.09 |

Concentrations are mgCB3988 per ml urine or bile and per g wet weight for the tissues analysed. %Dose is the % of the dose administered present in the total sample. ND, <0.02 mg g$^{-1}$ wet weight. Values are the mean ± standard deviation of data from 3–4 mice.

### *$^{19}$F-NMR spectroscopy of CB3988 in rats*

The time course for the appearance and decline of NMR signal from the upper abdomen of rats following the administration of 100 mg kg$^{-1}$ CB3988 is shown in Figure 6. Data for rats with intact and cannulated bile ducts are given and, as in the case of mice, all spectra contained only the peak associated with CB3988, no metabolites being detected. In both cannulated and bile duct-intact rats, NMR signal was detected within 2 min of the bolus dose of CB3988 with signal intensity reaching a maximum value by 10 min. Thereafter the signal declined rapidly, particularly in bile duct cannulated rats where the limit of detection was reached by 15–20 min and signal was not detected again during the remainder of the experiment (60 min). The half-lives for the decline in the NMR in the three bile duct cannulated rats were 6.5 ± 0.8, 6.9 ± 0.8 and 5.6 ± 2.9 min, overall mean 6.5 ± 0.8 min. In the rats with intact bile ducts the NMR signal did not decline as rapidly as in the cannulated rats and did not drop below the limit of detection during the experiment (0–60 min). Indeed, in two of the rats, from 30 min onwards the NMR signal had stabilised. These data suggest that the signal detected in rats with intact bile ducts is due in

**Figure 6** Time course for the appearance and decline of NMR signal in the upper abdomen of rats following 100 mg kg$^{-1}$ i.v. CB3988. Solid lines are rats with bile duct cannulae and broken lines are animals with intact bile ducts. Each line represents data from an individual rat.

**Figure 7** Plasma concentrations of CB3988 (O, Δ) and ICI 198583 (□) in rats following 100 mg kg$^{-1}$ i.v. Data for CB3988 are from rats with intact bile ducts (O) and those with cannulated bile ducts (Δ). Points are the mean of estimations in three rats for each treatment and bars the standard deviations of the means.

**Table II** Effect of bile duct cannulation and the 2'trifluoromethyl group on the pharmacokinetics of CB3988 in the rat (100 mg kg$^{-1}$ i.v.)

| *Compound* | *CB3988* | *CB3988* | *ICI 198583* |
|---|---|---|---|
| *Bile duct cannula* | – | + | + |
| $t_{1/2}$ alpha (min) | 4.1±1.0 | 3.3±0.8 | 6.1±0.9 |
| $t_{1/2}$ beta (min) | 22±4 | 27±12 | 30±8 |
| $V_1$ (ml kg$^{-1}$)[a] | 105±24 | 104±13 | 128±15 |
| $V_{dss}$ (ml kg$^{-1}$)[b] | 168±25 | 217±61 | 229±83 |
| Clearance (ml min$^{-1}$ kg$^{-1}$)[c] | 14.2±3.1 | 15.4±3.0 | 10.6±1.6 |
| Excretion (% dose, 0–60 min) | | | |
| Biliary | – | 77±7 | 75±8 |
| Urinary | 7.6±0.7 | 9.7±1.0 | ND |

Values are the mean ± s.d. from three rats for each treatment. ND, the 0–60 min urinary excretion for ICI 198583 was not determined, but the 0–240 min cumulative urinary excretion was 17.3±0.6% of the dose. [a]$V_1$, apparent volume of the central compartment. [b]$V_{dss}$, apparent volume of distribution at steady state. [c]Clearance, total plasma clearance.

part to drug re-entering the liver as part of the enterohepatic cycle. However, as shown in Figure 7 and Table II, there were no significant differences in the plasma levels or urinary excretion of CB3988 in rats with or without bile duct cannulae which probably reflects efficient uptake of CB3988 present in the portal system such that there is no passage of compound into the systemic circulation.

### *Comparison of the pharmacokinetics of CB3988 and ICI 198583 in rats*

To investigate the possibility that the 2'-trifluoromethyl group of CB3988 was a major determinant of the pharmacokinetics of the drug a comparison was made between CB3988 and its parent ICI 198583. The plasma levels of CB3988 and ICI 198583 are shown in Figure 7 and the pharmacokinetic parameters and urinary and bilary excretion data given in Table II. Inclusion of the 2'-trifluoromethyl group reduced the alpha phase half-live value ($t$ test, $P = 0.004$) and this led to levels of CB3988 being lower than those of ICI 198583, however, this was the only pharmacokinetic parameter for which there was a significant difference between CB3988 and ICI 198583.

### Discussion

The aim of the present study was to evaluate the utility of non-invasive $^{19}$F-NMR spectroscopy as a method of studying the pharmacokinetics of the antifolate CB3988 in mice and rats. This study is important both with regard to this particular class of compounds and because there is a general need to study non-invasive methods of pharmacokinetic monitoring in cancer chemotherapy. In both mice and rats NMR signal could be readily detected in the upper abdomen of the animal shortly after drug administration. Comparison of the NMR data with those of a conventional quantitative tissue distribution study performed using HPLC analysis demonstrated that the signal in the upper abdomen of mice was most probably derived from the gall bladder. Associated studies using whole body $^{19}$F-NMR imaging in mice following the administration of CB3988 confirm that the signal from the upper abdomen is derived from a discreet volume whose position is anatomically consistent with that of the gall bladder (Maxwell *et al.*, 1990). The other area examined in mice was the lower abdomen where signal was also readily detected. Again on the basis of HPLC analyses (Table I) and $^{19}$F-NMR imaging (Maxwell *et al.*, 1990) the source of the signal would appear to be primarily the urinary bladder. The time course of the appearance of the drug in the bladder indicates that the urinary excretion of the drug occurs rapidly and is essentially complete within 1 h (Figure 5). This is in marked contrast to the nephrotoxin CB3717 where there is delayed urinary excretion and drug still present in the kidney weeks after administration (Newell *et al.*, 1986). Thus NMR does appear to be capable of identifying rapid urinary elimination with this class of compound. In the light of clinical experience with CB3717 (Calvert *et al.*, 1986; Alison *et al.*, 1985) this information would be useful in the early clinical evaluation of CB3717 analogues.

In addition of urinary excretion, faecal elimination constitutes the other major route of excretion for CB3717 (Newell *et al.*, 1986) and its analogues (Newell *et al.*, 1988). Faecal elimination is preceded by biliary clearance with the majority of the dose present in the bile within 1 h in the case of both ICI 198583 and CB3988 (Table II). This is also the case for CB3717 itself at non-hepatotoxic doses (Newell & Siddik, unpublished results). Since biliary excretion is the major route of elimination, and the NMR signal from the upper abdomen of mice derives from the bile in the gall bladder, a relationship between the rate of decline in the plasma levels of CB3988 and the NMR signal might be anticipated. Although the plasma levels of CB3988 in mice were not determined as part of the present study, previous results with other C$^2$-desamino quinazolines indicate that the plasma half-life is approximately 20 min (Newell *et al.*, 1988). This value is consistent with that of 28 ± 6 min for the decline in the NMR signal reported herein. Thus for drugs which undergo extensive biliary elimination NMR studies of drug clearance from the liver may offer an indirect measure of plasma half-life. More generally, in those cases where drugs can be detected in organs of direct therapeutic or toxicological interest the results of NMR experiments may be more relevant than plasma analyses. For example, the relationship between drug levels in tumour tissue and activity should be better than that between plasma levels and activity.

Experiments were performed in rats to further investigate the relationship between CB3988 plasma half-life and the decline in NMR signal since it was technically possible to take blood samples, collect bile and perform spectroscopic experiments on the same animal. The experiments in bile duct cannulated rats, where enterohepatic circulation cannot occur, indicated that the alpha phase half-life for the clearance of CB3988 from plasma was 3.6 ± 0.3 min while the NMR signal from the upper abdomen decreased with a half-life of 6.5 ± 0.8 min. The similarity of these values again encourages the view that the signal from the liver can be used as an indirect measure of the plasma half-life of a drug, provided that the liver is the major organ for the clearance of the compound. When experiments were repeated in rats with an intact bile duct a more complex profile was obtained for the time course of NMR signal intensity from the upper abdomen (Figure 6). The difference between these results and those in bile duct cannulated animals strongly suggests an element of enterohepatic circulation in the disposition of the drug. Enterohepatic circulation has been observed in rats with CB3717 (Newell & Siddik, unpublished results) and methotrexate (Griffin & Said, 1987), and hence is not unexpected.

The rapid decline in the NMR signal from the liver of both mice and rats following CB3988 administration is again in contrast to the data obtained in mice (Newell *et al.*, 1986) and rats (Newell & Siddik, unpublished results) with CB3717. In both species reduced clearance of the drug from the liver was associated with hepatotoxicity. With $C^2$-desamino analogues of CB3717 both retention of drug in the liver and hepatotoxicity are absent (Newell *et al.*, 1988). However, these results were obtained using invasive techniques. The demonstration in the present study that it is possible to show rapid hepatic clearance non-invasively by NMR is again an encouraging result which would also be of value in the early clinical evaluation of CB3717 analogues.

The final point investigated in this study concerned the impart of the inclusion of the 2'-trifluoromethyl group on the pharmacokinetics of the antifolate molecule. In this respect the only significant effect of the 2'-trifluoromethyl group was to reduce the alpha phase half-life value with the result that plasma levels of CB3988 were lower than those of ICI 198583 (Figure 7). No other major qualitative or quantitative alterations in the distribution of the molecule, its clearance or its routes of elimination were observed.

However, it should be noted that associated biochemical studies have shown that the presence of the 2'-trifluoromethyl group reduces both the inhibitory activity of the compound towards the target enzyme, thymidylate synthase (7-fold), and the cytotoxicity of the agent (14-fold) in comparison to ICI 198583 (A.L. Jackman, unpublished results). Thus the potential problems of including the trifluoromethyl group as a 'label' in candidate drugs should not be underestimated and the use of a single fluorine atom may be more appropriate, but the resultant loss in sensitivity might compromise the utility of the methodology.

With regard to the general application of $^{19}$F-NMR in pharmacokinetic studies a number of points need to be considered. The major limitation of NMR is that the technique has only limited sensitivity. Thus in the present study signal detected in the upper and lower abdomen of mice was ascribed to drug present in the bile and urine at concentrations of 20–30 mg ml$^{-1}$ (40–60 mM). The dynamic range in these studies was approximately 10-fold and hence that limit of detection would be in the region of 4 mM. This figure could be reduced by the use of longer aquisition periods; however, in so doing time resolution would be lost which may be critical in the case of drugs with short half-lives. In contrast, conventional methods involving radiochemical and/or chromatographic analyses can provide information on drug levels in the low nM region. Thus, in the absence of marked improvements in sensitivity, *in vivo* $^{19}$F-NMR will only find application in those cases where relatively large doses of drug can be administered. This was possible in the case of CB3988 as $C^2$-desamino quinazoline antifolates can be administered to mice at 500 mg kg$^{-1}$ without producing any acute toxicity (Newell *et al.*, 1988). Since a number of antitumour agents are relatively impotent compounds, with clinical doses in the 100 mg to 1 g range, cancer chemotherapy represents an ideal area in which to assess the applications and limitations of *in vivo* $^{19}$F-NMR.

Despite the limitations of sensitivity with regard to *in vivo* spectroscopy, it is already clear that *ex vivo* analytical $^{19}$F-NMR does have considerable potential as a method to study drug and metabolite levels using biological fluids and isolated tissues. A number of studies have already shown the utility of the method in the case of the fluoropyrimidine drugs (Malet-Martino *et al.*, 1984, 1986; Keniry *et al.*, 1986; Vialaneix *et al.*, 1987; Hull *et al.*, 1988). The use of 'fluorine labels' in other anticancer drugs is clearly warranted and recent work with nitroimidazoles is a further example of this (Maxwell *et al.*, 1989). The studies with the fluoropyrimidines serve to highlight the major strength of NMR over radiochemical studies, namely, that the technique provides separate information on drug metabolites and the parent compound and not just on total drug derived material. This aspect of $^{19}$F-NMR was not apparent from the study reported in the present paper because quinazoline antifolates do not undergo extensive metabolism. Thus in the case of CB3717, the only extracellular metabolite which has been detected is the desglutamate compound which was found in the faeces and was shown to be a product of bacterial metabolism in the gastrointestinal tract (Newell *et al.*, 1986). Although polyglutamate metabolites of CB3717 have also been detected, both *in vitro* (Sikora *et al.*, 1988) and *in vivo* (Manteufel-Cymborowska *et al.*, 1986; Nair *et al.*, 1986), these compounds are thought to be rapidly catabolised once outside the cell. Within the cell polyglutamates are probably highly protein bound and would not have the freedom of motion required for a molecule to be detected by NMR. This requirement for molecules to be free to move in order to be detected by NMR spectroscopy may place an additional limitation on the detection of drugs in biological tissues since the line widths of NMR signals depends upon the rotational freedom of the nuclei involved. Thus the binding of a small molecule to a protein can result in line broadening to such an extent that some or all of the nuclei become 'NMR invisible'. Preliminary studies with CB3988 indicated that when the drug was added at 2 mM to a 50% (v/v) mouse liver homogenate the peak height signal to noise ratio was reduced 4-fold relative to an aqueous solution of the drug at the same concentration. Further experiments are required to define the extent to which this phenomenom will limit the application of *in vivo* NMR.

In conclusion, this study has demonstrated the utility of *in vivo* $^{19}$F-NMR spectroscopy as a method for studying the pharmacokinetics of a quinazoline antifolate drug in mice and rats. The results obtained by NMR indicate that CB3988 is cleared rapidly by both biliary and urinary elimination. These data are consistent with the lack of liver and kidney toxicity found with the $C^2$-desamino CB3717 analogues. Comparison of the NMR data with data obtained using conventional HPLC methodology indicates that the NMR signals detected in the upper and lower abdomen of mice are derived from the gall bladder and urinary bladder, respectively. Pharmacokinetic analyses in both mice and rats suggest that the rate of decline of the NMR signal derived from the upper abdomen may reflect the rate of drug clearance from the plasma and that in rats NMR can detect compound undergoing enterohepatic circulation. Comparison of the pharmacokinetics of CB3988 and ICI 198583 in rats indicated that the inclusion of the 2'trifluoromethyl group reduced the alpha phase half life, however, it did not markedly alter the rate or route of drug clearance. In view of the non-hazardous nature of *in vivo* NMR spectroscopy the studies reported herein are directly applicable to clinical investigations with the potential to provide hitherto unobtainable information.

This work was supported by grants from the Cancer Research Campaign and Medical Research Council. The authors are grateful to Professor A.H. Calvert for his interest and to Dr T.R. Jones for his encouragement during the planning stages of this project.

### References

ALISON, D.L., NEWELL, D.R., SESSA, C. & 4 others (1985). The clinical pharmacokinetics of the novel antifolate N$^{10}$-propargyl-5,8-dideazafolic acid (CB3717). *Cancer Chemother. Pharmacol.*, **14**, 265.

CALVERT, A.H., ALISON, D.L., HARLAND, S.J. & 9 others (1986). A phase 1 evaluation of the quinazoline antifolate thymidylate synthase inhibitor, N$^{10}$-propargyl-5,8-dideazafolic acid. *J. Clin. Oncol.*, **4**, 1245.

COLLINS, J.M., ZAHARKO, D.S., DEDRICK, R.L. & 1 other (1986). Potential roles for clinical pharmacology in phase 1 clinical studies. *Cancer Treat. Rep.*, **70**, 73.

EORTC PHARMACOKINETICS AND METABOLISM GROUP (1987). Pharmacokinetically guided dose escalation in phase 1 clinical studies. Commentary and proposed guidelines. *Eur. J. Cancer Clin. Oncol.*, **23**, 1083.

GRIFFIN, D. & SAID, H.M. (1987). The enterohepatic circulation of methotrexate in vivo: Inhibition by bile salt. *Cancer Chemother. Pharmacol.*, **19**, 40.

HOUSTON, J.B. (1985). Kinetics of drug metabolism and disposition: physiological determinants. In *Drug Metabolism and Disposition: Considerations in Clinical Pharmacology*, Wilkinson, G.R. & Rawlins, M.D. (eds). p. 63. MTP Press: Lancaster.

HUGHES, L.R. (1986). Preparation of N-(((3,4-dihydro-4-oxo-6-quinazolinyl) methyl) amino) aroyl amino acids as antitumour agents. GB Patent Appl. 86/7,683.

HUGHES, L.R., MARSHAM, P.R., OLDFIELD, J & 5 others (1988). Thymidylate synthase (TS) inhibitory and cytostatic activity of a series of $C^2$ substituted -5,8-didezafolates. *Proc. Am. Assoc. Cancer Res.*, **29**, 286.

HULL, W.E., PORT, R.E., HERRMANN, R. & 2 others (1988). Metabolites of 5-fluorouracil in plasma and urine, as monitored by $^{19}F$ nuclear magnetic spectroscopy, for patients receiving chemotherapy with or without methotrexate pretreatment. *Cancer Res.*, **48**, 1680.

JACKMAN, A.L., TAYLOR, G.A., MORAN, R. & 6 others (1988). Biological properties of 2-desamino-2 substituted -5,8-dideazafolates that inhibit thymidylate synthase (TS). *Proc. Am. Assoc. Cancer Res.*, **29**, 287.

JENNRICH, R.I. & SAMPSON, P.F. (1968). Application of a stepwise regression to non-linear least squares estimation. *Technometrics*, **10**, 63.

KENIRY, M., BENZ, C., SHAFER, R.H. & 1 other (1986). Noninvasive spectroscopic analysis of fluoropyrimidine metabolism in cultured tumour cells. *Cancer Res.*, **46**, 1754.

MALET-MARTINO, M.-C., MARTINO, R., LOPEZ, A. & 4 others (1984). New approach to metabolism of 5'deoxy-5-fluorouridine in humans with fluorine-19 NMR. *Cancer Chemother. Pharmacol.*, **13**, 31.

MALET-MARTINO, M.-C., FAURE, F., VIALANEIZ, J.-P. & 3 others (1986). Non-invasive fluorine-19 NMR study of fluoropyrimidine metabolism in cultures of human pancreatic and colon adenocarcinomas. *Cancer Chemother. Pharmacol.*, **18**, 5.

MANTEUFFEL-CYMBOROWSKA, M., SIKORA, E. & GRZELAKOWSKA-SZTABERT, B. (1986). Polyglutamylation of the antifolate anticancer drug $N^{10}$-propargyl-5,8-dideazafolic acid (CB3717) in the mouse. *Anticancer Res.*, **6**, 807.

MARSHAM, P.R., JACKMAN, A.L., HUGHES, L.R. & 6 others (1990). Quinazoline antifolate thymidylate synthase inhibitors: benzoyl ring modifications in the $C^2$-methyl series. *J. Med. Chem.* (in the press).

MAXWELL, R.J., WORKMAN, P. & GRIFFITHS, J.R. (1989). Demonstration of tumor-selective retention of fluorinated nitroimidazole probes by $^{19}F$ magnetic resonance spectroscopy *in vivo*. *Int. J. Radiat. Oncol. Biol. Phys.*, **16**, 925.

MAXWELL, R.J., FRENKIEL, T.A, NEWELL, D.R. & 2 others (1990). $^{19}F$-NMR imaging of drug distribution *in vivo*: the disposition of an antifolate anticancer drug in mice. *Magn. Reson. Med.* (in the press).

NAIR, M.G., METHA, A.P. & NAIR, I.G. (1986). The metabolism of $N^{10}$-(propargyl) 5,8-dideazafolic acid (PDDF) in mice. *Fedn. Proc.*, **45**, 821.

NEWELL, D.R., ALISON, D.L., CALVERT, A.H. & 5 others (1986). Pharmacokinetics of the thymidylate synthase inhibitor $N^{10}$-propargyl-5,8-dideazafolic acid (CB3717) in the mouse. *Cancer Treat. Rep.*, **70**, 971.

NEWELL, D.R., MAXWELL, R.J., GRIFFITHS, J.R. & 3 others (1988). Pharmacokinetic and toxicity studies with $C^2$-desamino-$C^2$-substituted analogues of $N^{10}$-propargyl-5,8-dideazafolic acid (CB3717). *Proc. Am. Assoc. Cancer Res.*, **29**, 286.

NEWELL, D.R. (1989). Pharmacokinetic determinants of the activity and toxicity of antitumour agents. *Cancer Surv.*, **8**, 557.

SELINSKY, B.S., THOMPSON, M. & LONDON, R.E. (1987). Measurement of *in vivo* hepatic halothane metabolism using $^{19}F$-NMR spectroscopy. *Biochem. Pharmacol.*, **36**, 413.

SELINSKY, B.S., PERLMAN, M.E. & LONDON, R.E. (1988*a*). *In vivo* nuclear magnetic resonance studies of methoxyflurane metabolism. I. Verification and quantitation of methoxydifluoroacetate. *Mol. Pharmacol.*, **33**, 559.

SELINSKY, B.S., PERLMAN, M.E. & LONDON, R.E. (1988*b*). *In vivo* nuclear magnetic resonance studies of hepatic methoxyflurane metabolism. II. A re-evaluation of hepatic metabolic pathways. *Mol. Pharmacol.*, **33**, 567.

SIKORA, E., JACKMAN, A.L., NEWELL, D.R. & 1 other (1988). Formation and retention and biological activity of $N^{10}$-propargyl-5,8-dideazafolic acid (CB3717) polyglutamates in L1210 cells *in vitro*. *Biochem. Pharmacol.*, **37**, 4047.

SIDDIK, Z.H. & NEWELL, D.R. (1988). Radiochemicals in cancer research and clinical oncology. In *Radiochemicals in Biomedical Research*, Evans, E.A. & Oldham, K.G. (eds) p. 118. John Wiley and Sons: Chichester.

STEVENS, A.N., MORRIS, P.G., ILES, R.A. & 2 others (1984). 5-Fluorouracil metabolism monitored *in vivo* by $^{19}F$ NMR. *Br. J. Cancer*, **50**, 113.

VIALANEIX, J.P., MALET-MARTINO, M.C., HOFFMANN, J.S. & 2 others (1987). Direct detection of new flucytosine metabolites in human biofluids by $^{19}F$ nuclear magnetic resonance. *Drug Metab. Disp.*, **15**, 718.

WOLF, W., ALBRIGHT, M.J., SILVER, M.S. & 3 others (1987). Fluorine-19 NMR spectroscopic studies of the metabolism of 5-fluorouracil in the liver of patients undergoing chemotherapy. *Magn. Reson. Imaging*, **5**, 165.

WYRWICZ, A.M., CONBOY, C.B., RYBACK, K.R. & 2 others (1987). *In vivo* $^{19}F$-NMR study of isoflurance elimination from brain. *Biochim. Biophys. Acta*, **927**, 86.